# Report of an unusual combination of arterial, venous and neural variations in a cadaveric upper limb

**DOI:** 10.1186/1749-7221-9-2

**Published:** 2014-02-05

**Authors:** Theodore G Troupis, Adamantios Michalinos, Vasiliki Manou, Dimitrios Vlastos, Elizabeth O Johnson, Theano Demesticha, Panayiotis Skandalakis

**Affiliations:** 1 Department of Anatomy, Faculty of Medicine, National and Kapodistrian University of Athens, Athens, Greece

**Keywords:** Brachial plexus, Brachial artery, Cephalic vein

## Abstract

In this study an unusual combination of arterial, venous and neural variations discovered during dissection of cervical, axillary and brachial area of a cadaver is described. Variations are thoroughly described and literature is briefly reviewed. Lateral cord of brachial plexus was not formed; Eight Cervical root divided into anterior and posterior division before uniting with First Thoracic root and Upper Trunk was unusually short. Axillary artery gave origin to a superficial brachial artery and then continued as deep brachial artery. Multiple variations in typical axillary artery branches were present including existence of inferior pectoral artery. Cephalic vein was absent. A variety of interventions, from relative simple as central venous catheter placement to most complicated as brachial plexus injury repair demand thorough knowledge of area’s regional anatomy. Familiarity with anatomic variations allows more precise and careful interventions. Research on these variations is valuable for anatomists and embryologists but also for clinicians because it may provide useful information for non - typical cases but also helps in raising a high level of suspicion.

## Background

Anatomy of brachial plexus and axillary artery (AA) is complicated, subjected to a wide variety of variations and of major importance for many pathological and surgical conditions. Embryologic explanation of its development is difficult. Clinical sequelae of variations in the area are often unpredictable but of major importance. Many authors [1-4] have studied and provided analytical descriptions of Brachial Plexus, AA and their relationship.

In this study an unusual combination of arterial, venous and neural variations concerning axillary and brachial region is described. Variations are analyzed both separately and as a pattern and examined in the light of their clinical and embryological significance. Classification with reference to widely used classification systems of the area is also attempted. Literature is briefly reviewed and anatomical, embryological and clinical questions raised are discussed.

### Case presentation

During dissection of the neck, right axillary and brachial region of a male cadaver at the Laboratory of Anatomy, Faculty of Medicine, National and Kapodistrian University of Athens, Greece an unusual combination of arterial, venous and neural variations was identified. Primary dissection was performed by undergraduate students and further dissection by authors. Variations were described, photographed and measured.

Brachial plexus took origin from C5-T1 roots. No contribution of C4 or T2 roots was encountered. The C5 and C6 roots united in an unusually short Upper Trunk (UT) which immediately divided into its anterior division (AD [UT]) and posterior division (PD [UT]). Suprascapular nerve took origin from UT. The C7 root continued typically as Medial Trunk (MT) and divided into its anterior (AD [MT]) and posterior division (PD [MT]). The C8 root divided into anterior and posterior divisions (PD [C8]) before uniting with T1 root. Anterior division of C8 root united with T1 root and together formed Inferior Trunk (IT). The AD [UT] and AD [MT] did not unite thus no Lateral Cord (LC) was formed. The AD [UT] divided into Musculocutaneous Nerve (MCN) and lateral root of median nerve (LR [MN]). The AD [MT] gave off lateral pectoral nerve and then united with LR [MN]. The IT, which since C8 root had divided before union with T1 is also AD [IT], continued into medial cord as usually described and later divided into medial root of median nerve (MR [MN]) and Ulnar Nerve (UN). Medial Brachial Cutaneous Nerve (MBCN) was one of its branches. The PD [C8] united with PD [MT] and these two united later with PD [UT] so to form posterior cord (PC). The MCN was formed by AD [UT] and thus did not contain C7 fibers while Median Nerve (MN) and UN were formed as usual. The PC did not contain T1 fibers. After this level, nerves continued their course and distribution normally in humerus, forearm and hand. No other variations of the branches of the brachial plexus were encountered.

Transverse cervical artery passed above all branches of the brachial plexus. Suprascapular and deep scapular artery passed between AD [UT] and MT. No other variations concerning neural elements of humeral, axillary and neck region were encountered.

**Figure 1:**
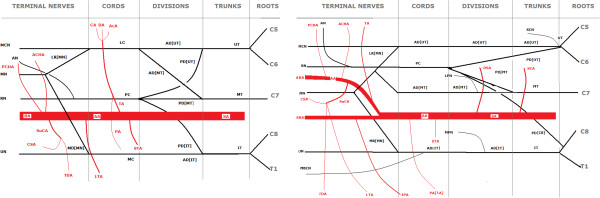
**Schematic representation of brachial plexus and artery in normal and variant form.** UT: Upper trunk; MT: Medial Trunk; IT: Inferior Trunk, AD [UT]: Anterior division of Upper Trunk; AD [MT]: Anterior division of Middle Trunk; AD [IT]: Anterior division of Inferior Trunk; PD [UT]: Posterior division of Upper Trunk; PD [MT]: Posterior division of Middle Trunk; PD [IT]: Posterior division of Inferior Trunk; PD [C8]: Posterior division of C8; MC Medial Cord; PC: Posterior cord; MR [MN]: Middle root of median nerve; LR [MN]: Lateral root of median nerve; MCN: Musculocutaneous Nerve; MN: Median Nerve; UN: Ulnar Nerve; RN: Radial Nerve; AN: Axillary Nerve; SCN: Suprascapular Nerve; MBCN: Medial Brachial Cutaneous Nerve; LPN: Lateral pectoral nerve; MPN: Medial Pectoral Nerve; SA: Subclavian Artery; AA: Axillary Artery; SBA: Superficial Brachial Artery; dBA: Deep Brachial Artery; DSC: Deep Scapular Artery; SCA: SupraScapural Artery; STA: Superior Thoracic Artery; TA: Thoracoacromial Artery; CA: Clavicular Artery; DA: Deltoid Artery; AcA: Acromial Artery; SuSA: SubScapular Artery; LTA: Lateral Thoracic Artery; TDA: Thoracodorsal Artery; CSA: Circumflex Scapular Artery; ACHA: Anterior Circumflex Humeral Artery; PCHA: Posterior Circumflex Humeral Artery; IPA: Inferior Pectoral Artery.

**Figure 2:**
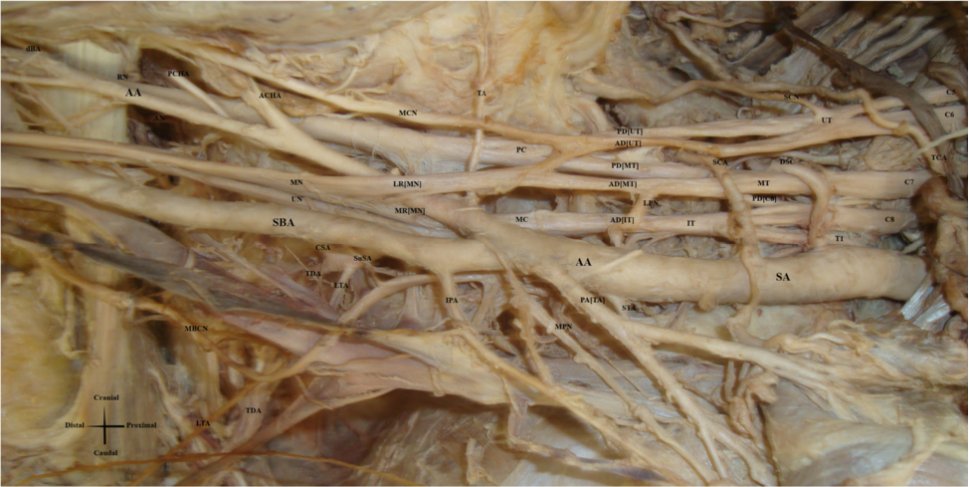
**Cadaveric photo of variation of brachial plexus and axillary artery variations.** UT: Upper trunk; MT: Medial Trunk; IT: Inferior Trunk; AD [UT]: Anterior division of Upper Trunk; AD [MT]: Anterior division of Middle Trunk; AD [IT]: Anterior division of Inferior Trunk; PD [UT]: Posterior division of Upper Trunk; PD [MT]: Posterior division of Middle Trunk; PD [C8]: Posterior division of C8; MC: Medial Cord; PC: Posterior cord; MR [MN]: Middle root of median nerve; LR [MN: Lateral root of median nerve; MCN: Musculocutaneous Nerve; MN: Median Nerve; UN: Ulnar Nerve; RN: Radial Nerve; AN: Axillary Nerve; SCN: Suprascapular Nerve; MBCN: Medial Brachial Cutaneous Nerve; LPN: Lateral pectoral nerve; MPN: Medial Pectoral Nerve; SA: Subclavian Artery; SCA: Suprascapular Artery; DSC: Deep Scapular Artery; AA: Axillary Artery; SBA: Superficial Brachial Artery; dBA: Deep Brachial Artery; DSC: Deep Scapular Artery; TCA: Transverse Cervical Artery; STA: Superior Thoracic Artery; TA: Thoracoacromial Artery; PA [TA] Pectoral Branch of Thoracoacromial Artery; SuSA: Subscapular Artery; LTA: Lateral Thoracic Artery; TDA: Thoracodorsal Artery; CSA: Circumflex Scapular Artery; IPA: Inferior Pectoral Artery; ACHA: Anterior Circumflex Humeral Artery; PCHA: Posterior Circumflex Humeral Artery.

The AA at the level of the union of the two root of the MN, also known as ansa medianis, divided into two branches. Superior Thoracic Artery (STA) occurred and coursed normally. Pectoral artery of Thoracoacromial Artery (PA [TA]) occurred before the division. After the division, two branches occurred, one that continued as AA and another that passed superficial to Ansa Medianis. The AA gave off the other branches of Thoracoacromial Artery (TA), namely clavicular, deltoid and acromial and also Subscapular artery (SuCA) that later trifurcated into lateral thoracic artery (LTA), Circumflex Scapular Artery (CSA) and Thoracodorsal Artery (TDA). Then it gave origin to Anterior Circumflex Humeral Artery (ACHA), Posterior Circumflex Humeral Artery (PCHA) and ended as deep brachial artery (dBA). The other branch continued superficial to MR [MN], then in front of MN and finally reached the normal position and course of Brachial Artery at about the middle of the humerus. Taking in consideration its origin, course and branches we considered it a Superficial Brachial Artery (SBA). Immediately after SBA’s branching point, it gave a small branch that coursed towards inferior surface of pectoralis major muscle and thus corresponded to inferior pectoral artery (IPA). Apart from that it gave origin to only muscular branches and bifurcated into Radial and Ulnar Artery. A highly diagrammatic presentation of typical anatomy is provided at Figure 1 and that of the variation is illustrated at Figure 1 while a photo at Figure 2. No other variations concerning arterial system were encountered at axillary, branchial and forearm region.

**Figure 3:**
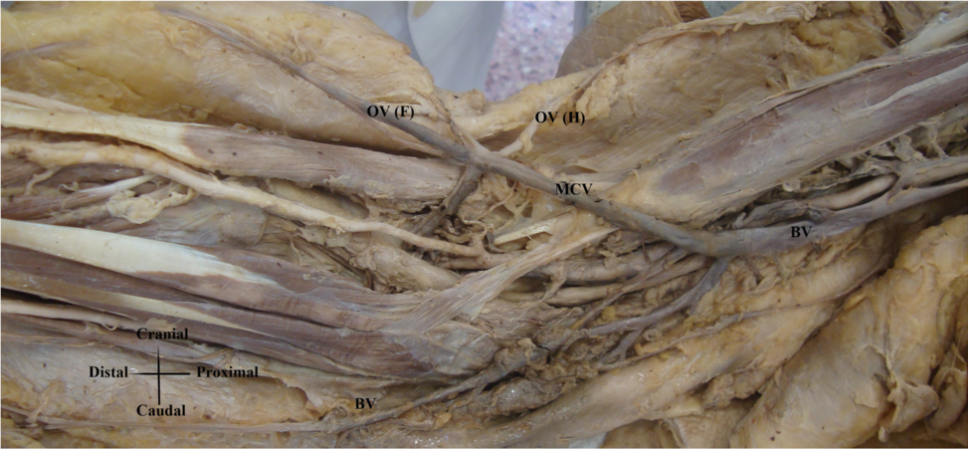
**Cadaveric photo of cephalic vein abscence.** BV: Basilic Vein; MCV: Middle cubital vein; OV (F): Oblique vein draining forearm; OV (H): Oblique vein draining humeral region.

The Cehpalic Vein (CV) was absent. Superficial radial surface of the forearm and brachial region was drained by two veins that had oblique course, upwards and downwards respectively, and ended as branches of the basilica vein (Figure 3).

Some of these variations have already been described separately. However, to the best of our knowledge, there has never been described such a combination of arterial and neural variations, not to mention the absence of the CV.

## Discussion

Variations in brachial plexus formation are common and complicated. Their frequency reaches 54% and their degree of complexity is such that makes every systematization effort very difficult [5]. In 1918 Kerr [1] in an attempt to classify brachial plexus variations organized them in 3 groups and 7 subgroups with respect to contribution of C4 and T2 root (groups) and participation of roots in the formation of cords (subgroups). Taking in consideration these characteristics, pattern described here corresponds to type E, i.e. contribution of C5, C6, C7, C8 and T1 roots with LC “formed” from C5, C6 and C7 root and medial cord from C8 and T1 roots. (29.14%). He also described the frequency of certain variations in brachial plexus. Extending further his investigation, he described a number of rare variations corresponding to brachial plexus elements arrangement and described 29 interesting and difficult to classify cases. According to Kerr non - formation of LC has a frequency of 3%. The C8 root dividing before uniting with T1 root has a frequency of 3.5% and union of posterior division of Inferior Trunk and PD [MT] before union with PD [UT] accounts for 14%. The latter two variations are combined in only 1 case in Kerr’s research. Taking in consideration all 3 cases, our case does not correspond precisely to any of Kerr’s cases, not accounting arterial and venous variations. Other authors [6] agree that non formation of LC accounts for 3%. Various researchers investigated morphometry of brachial plexus. Significance of such measurements is difficult to evaluate due to arrangement of connective and neural tissue [1] and of uncertain clinical importance.

Precise embryologic explanation of brachial plexus variations is not fully understood. It is known that embryonic somites, carrying their innervation, migrate to the limbs and Brachial Plexus is formed latter. In fetal life brachial plexus appears as a radicular cone that latter divides into ventral and dorsal branches [7]. Formation of brachial plexus is interconnected with axillary artery formation and variations between them coexist and interfere, as in our case. Variations of brachial plexus and axillary artery coexist in at least 8% of the cases. Occurrence of axillary artery from 6th or 8th intersegmental artery or variations in its branches is of importance for brachial plexus final form although the exact way remains obscure [2].

Functional significance of brachial plexus variations is not known either. Taking in consideration its embryologic formation, especially the fact that innervation of somites by neurotomes is established before formation of brachial plexus they might are not always important. In our case the only evident change is lack of C7 root innervation at MCN and lack of T1 root innervation at PC. Brachial plexus is formed by an extremely large number of fascicles that combine and recombine as we proceed more distal. Analysis at microscopic level has proved that C7 contribution at MCN and T1 at PC is minimal or absent. However variations of brachial plexus, even if they do not alter terminal nerves, can be useful in explaining various neuropathies or neurological deficits [4,5,7]. It has been stated that a number of variations of UT in its formation pattern, course and relative position to anterior scalene muscle might predispose to appearance of thoracic outlet syndrome [8].

Occurrence of arteries near or between elements of brachial plexus is high reaching 23% [9]. In our case an interesting combination of upper limb’s variations was observed. First the existence of SBA as a branch of AA was observed, giving off as only branch the IPA Also the LTA originated from SuCA. Last but not least the TA was absent and its branches arose directly from the second part of the axillary artery. The most frequent anatomic variations of the axillary artery are the persistent SBA, high division of the brachial artery, radial artery and ulnar artery [10-13]. SBA is a common variation. Lippert and Pabst calculate it at about 22% but especially the SBA deriving from the AA only at 4% [14]. As for the LTA originating with the SuSA artery it is also a quite common variation accounting for 10% [14]. Kodama’s study noticed a frequency of IPA originating from SBA approximately at 8,7%. Whenever or not the SBA is combined with the IPA existence, it is noteworthy that the latter artery never happens to ramify from typical brachial artery which lies deep to the median nerve. Same mechanisms that induce its existence might also induce SBA’s existence. Moreover he suggested that the IPA might be a derivative of or a trigger for SBA morphogenesis [15]. Finally, absent TA and origin of its branches directly from the AA is described with a frequency of about 11.25% [16].

When mentioning the upper limb, in particular the vascular system and its variations, it is necessary to discuss the embryology of the region. Originally the Subclavian Artery (SA) extends to the wrist, where it terminates by dividing into terminal branches for the fingers, later becoming interosseous and median artery. In embryos 21 mm long the SBA develops in the axillary region and traverses the medial surface of the arm ending to the posterior surface of the wrist. When the embryo reaches the length of about *23* mm, the median artery undergoes retrogression, becoming a small, slender structure. The SBA is a consistent embryonic vessel that plays an important role in the normal arterial morphogenesis of the upper limb. At the elbow, an anastomotic branch between the brachial artery and the SBA becomes enlarged sufficiently to form the distal portion of the latter, the radial artery. Radial artery soon becomes a major artery of the forearm while the proximal portion of the SBA atrophies correspondingly.

Every vascular variation can be traced back to an embryological origin. Jurjus [10] mentioned six explanations for the variations observed:

1. The choice of unusual paths in the primitive vascular plexus.

2. The persistence of vessels which are normally obliterated.

3. The disappearance of vessels which are normally retained.

4. An incomplete development.

5. The fusion and absorption of parts which are normally distinct.

6. A combination of factors leading to an atypical pattern normally encountered.

Superficial surface of the forearm is drained by CV and basilica vein, united by median cubital vein. The CV ascends in front of the elbow superficial to a groove between the brachioradialis and biceps, crosses superficial to the lateral cutaneous nerve of the forearm, ascends lateral to biceps and between pectoralis major and deltoid, where it adjoins the deltoid branch of the TA. Entering the infraclavicular fossa it passes behind the clavicular head of pectoralis major, it pierces the clavipectoral fascia, crosses the AA and joins the axillary vein just below clavicular level. It may connect with the external jugular vein by a branch crossing anterior to the clavicle [14,17].

Numerous variations in the course and terminal drainage of the cephalic vein have been described including persistence of the jugulocephalic vein [18], absence of cephalic vein [19-23], cephalic vein draining into the external or internal jugular vein, cephalic vein draining into the subclavian vein or into the junction of subclavian and internal jugular vein, cephalic vein draining into the basilic vein [24] and accessory cephalic vein [25]. Concerning frequency of the absence of the cephalic vein, Loukas et al. [19] report 5% Le Saout et al. [20] report 19.7%, De Rosa [21] et al. report 5.3% and Yeri L.A. et al. [22] report 8.2%.

During embryonic life principle arm vein remains in caudal or ulnar positions and joins the lateral thoracic (thoracoepigastric) vein to form the primitive subclavian trunk. A small cranial tributary of this trunk develops into a primitive cephalic vein and follows the radial border of the arm, becoming continuous with the ulnar vein by way of the marginal vein outlining the hand plate. The proximal end of this new vein, namely the cephalic vein, curves cranially around the superficial aspect of the primordium of the clavicle. This arched part of the vessel has been called the jugulocephalic vein [18]. Sometimes the median cubital vein is large, transferring most blood from the cephalic to the basilica vein; the proximal cephalic vein is then absent- as in our case- or much diminished [26].

The clinical importance of the described axillary variation is of utmost significance for surgeons, cardiologists and vascular specialists but also for diagnostic reasons. It is specially relevant in cases of arteriovenous fistulae, aneurysms and abscesses drainage in region of axilla, arm and cubital fossa [27]. In angiographic studies preceding coronary artery bypass surgery such aberrant anatomy should be timely confirmed to reduce the incidence of iatrogenic injuries. An abnormal SBA as in this case may be mistaken for basilic vein during cannulation [28,29]. If certain drugs are injected into these vessels, the result might be disastrous: gangrene with subsequent partial or total loss of the hand [28]. Axillary and brachial artery variations are relevant in shoulder, arm and forearm surgery, in fractures and dislocations. What is more, diagnostically this type of variation may disturb the evaluation of angiographic images. Awareness of such abnormal axillary vasculature is crucial in use of superficial brachial artery flap in plastic surgery and protection of axillary artery in breast cancer surgery. Finally, accurate knowledge of the relationships and course of these major arterial conduits, and particularly of their variation patterns, is of considerable practical importance in the conduct of reparative surgery in the arm, forearm, and hand. Otherwise, less critical knowledge of anatomy might lead to hazards in surgery of the upper extremity [30].

Central venous access via the upper extremity veins is used in various procedures because it is easy to perform, convenient for the patients and has low complications rate. The relatively low mobility of the central veins of the upper extremity and the neck also affords low mechanical stress on the indwelling hardware. The cephalic vein cutdown is reported to has low complication rates particularly when compared with subclavian vein cannulation. Also cephalic vein cutdown poses no risk of pneumothorax. This technique is widely used for the placement of pacing and defibrillation leads and chronic indwelling venous catheters. It is obvious that the absence of the cephalic vein would obviate the need for an approach through a more proximal vein (axillary, subclavian) to obtain central venous access, a technique that entails comperatively increased morbidity rates. [31] Currently there are neither simple diagnostic techniques for the location of such persons nor specific indications for the use of more sophisticated ones (eg, phlebography) thus a high rate of clinical suspicion is necessary in case of intervention need in cephalic vein area.

## Conclusions

Deep knowledge of anatomy of Brachial Plexus is necessary for an extremely large number of procedures including direct trauma, fractures, obstetrical wounds and a wide variety of neuropathies. Methods of preoperative evaluation of brachial plexus include electromyography, somatosensory evoked potentials myelography, computerized tomography, myelo Computerized Tomography, magnetic resonance imaging, and magnetic resonance neurography. Profound knowledge is also necessary for success of regional anaesthesia with ultrasound guidance [9] Although not all variations can be encountered, knowledge of the possibility of their existence raised a high level of suspicion that helps neurologists, orthopaedic surgeons, surgeons and neurosurgeons avoid unnecessary mistakes and thus provide best possible care and ensure patients’ interests.

### Consent

Since this is a cadaveric study, no special consent form is necessary.

## Abbreviations

MT: Medial trunk; UT: Upper trunk; IT: Inferior trunk; AD [UT]: Anterior division of upper trunk; AD [MT]: Anterior division of middle trunk; AD [IT]: Anterior division of inferior trunk; PD [UT]: Posterior division of upper trunk; PD [MT]: Posterior division of middle trunk; PD [IT]: Posterior division of inferior trunk; PD [C8]: Posterior division of C8; MC: Medial cord; PC: Posterior cord; MR [MN]: Middle root of median nerve; LR [MN]: Lateral root of median nerve; MCN: Musculocutaneous nerve; MN: Median nerve; UN: Ulnar nerve; RN: Radial nerve; AN: Axillary nerve; SCN: Suprascapular nerve; MBCN: Medial brachial cutaneous nerve; LPN: Lateral pectoral nerve; MPN: Medial pectoral nerve; SA: Subclavian artery; AA: Axillary artery; SBA: Superficial brachial artery; dBA: deep brachial artery; DSC: Deep scapular artery; SCA: SupraScapural artery; STA: Superior thoracic artery; TA: Thoracoacromial artery; CA: Clavicular artery; DA: Deltoid artery; AcA: Acromial artery; SuSA: Subscapular artery; LTA: Lateral thoracic artery; TDA: Thoracodorsal artery; CSA: Circumflex scapular artery; ACHA: Anterior circumflex humeral artery; PCHA: Posterior circumflex humeral artery; IPA: Inferior pectoral artery; BV: Basilic vein; MCV: Middle cubital vein; OV (F): Oblique vein draining forearm; OV (H): Oblique vein draining humeral region.

## Competing interests

The authors declare that they have no competing interests.

## Authors’ contribution

TT had the study concept and design. AM participated in dissection, analysis and interpretation of data. VM participated in dissection, analysis and interpretation of data. DV participated in dissection, analysis and interpretation of data. EOJ participated in drafting of the manuscript. PS had the Critical revision of the manuscript for important intellectual content. All authors’ read and approved the final manuscript.
